# Mechanism of Myoglobin Molecule Adsorption on Silica: QCM, OWLS and AFM Investigations

**DOI:** 10.3390/ijerph18094944

**Published:** 2021-05-06

**Authors:** Monika Wasilewska, Małgorzata Nattich-Rak, Agata Pomorska, Zbigniew Adamczyk

**Affiliations:** Jerzy Haber Institute of Catalysis and Surface Chemistry, Polish Academy of Science, Niezapominajek 8, 30-239 Cracow, Poland; malgorzata.nattich-rak@ikifp.edu.pl (M.N.-R.); agata.pomorska@ikifp.edu.pl (A.P.); zbigniew.adamczyk@ikifp.edu.pl (Z.A.)

**Keywords:** adsorption of myoglobin, myoglobin layers, myoglobin zeta potential, OWLS measurements, QCM measurements, silica sensor, zeta potential of myoglobin

## Abstract

Adsorption kinetics of myoglobin on silica was investigated using the quartz crystal microbalance (QCM) and the optical waveguide light-mode spectroscopy (OWLS). Measurements were carried out for the NaCl concentration of 0.01 M and 0.15 M. A quantitative analysis of the kinetic adsorption and desorption runs acquired from QCM allowed to determine the maximum coverage of irreversibly bound myoglobin molecules. At a pH of 3.5–4 this was equal to 0.60 mg m^−2^ and 1.3 mg m^−2^ for a NaCl concentration of 0.01 M and 0.15 M, respectively, which agrees with the OWLS measurements. The latter value corresponds to the closely packed monolayer of molecules predicted from the random sequential adsorption approach. The fraction of reversibly bound protein molecules and their biding energy were also determined. It is observed that at larger pHs, the myoglobin adsorption kinetics was much slower. This behavior was attributed to the vanishing net charge that decreased the binding energy of molecules with the substrate. These results can be exploited to develop procedures for preparing myoglobin layers at silica substrates of well-controlled coverage useful for biosensing purposes.

## 1. Introduction

Adsorption of proteins at various surfaces is a prerequisite of their separation by filtration and chromatography, for biosensing, bioreactors, tissue culture and immunological assays [1,2,3,4]. Because of its vital significance, this phenomenon has been extensively studied using numerous experimental techniques and theoretical approaches [5,6,7,8,9,10,11,12,13,14]. However, despite this effort there still persist controversies concerning important aspects of protein adsorption, such as the nature of driving forces, reversibility of this process, the extent of molecule conformational changes, etc. [6]. 

In an attempt to unveil protein adsorption mechanisms, we focus attention in this work on myoglobin making up 0.3–10% of the muscle mass of terrestrial and diving mammals, respectively [15,16]. It is a globular protein characterized by an ellipsoidal shape consisting of a single polypeptide chain (153 amino acids, eight helices) and a single iron protoporphyrin or heme moiety and is thus classified as a metalloprotein. Its molar mass derived from chemical composition is equal to 17,800 g mol^−1^, which was confirmed by neutron scattering measurements [17], and the density is equal to 1.35 g cm^−3^ [18]. Other properties and functions of myoglobin have been investigated in ref. [19].

Myoglobin is responsible for oxygen storage and transport to muscles [18,20,21,22,23]. It also serves as an efficient biomarker of acute myocardial infections, cardiac injury and renal failure [24]. 

Because of its important biological role, the adsorption of myoglobin on various surfaces was studied usually with the aim of preparing biosensors. In ref. [25], the immobilization of myoglobin and hemoglobin using the matrix-assisted pulsed laser evaporation (MAPLE) technique on a surface plasmon resonance transducer was investigated. Protein immobilization was characterized by Fourier transform infrared spectroscopy (FTIR), transmission electron microscopy (TEM) and atomic force microscopy (AFM). It was shown that the protein films exhibited suitable bioactivity. The obtained protein films allowed ultra-sensitive detection of glucose.

In ref. [26], myoglobin was immobilized on sodium alginate films to produce oxygen facilitated transport membranes, exploiting the fact that it is a single-site oxygen biocarrier. Yamaguchi A. et al. [27,28] studied the adsorption of myoglobin at mesoporous silica using the differential scanning calorimetry and the optical absorption spectroscopy methods in order to determine the role of pH and pore size. 

In ref. [29], the adsorption of myoglobin, bovine serum albumin and thyroglobulin on titania layers sputtered on silica was studied at pH 7.4 by ellipsometry. Using AFM ambient air imaging, both the rms, the skewness and surface kurtosis of the substrate surfaces were determined and correlated with the adsorption kinetics and the protein film thickness. 

The influence of myoglobin concentration on the structure of its layers at different hydrophobic surfaces (polystyrene and octadecyltrichlorosilane) was studied in ref. [30] by neutron reflectometry. Because the protein layers exhibited hydrophilic properties, it was postulated that myoglobin molecules are attached to the substrates by their hydrophobic parts and expose the hydrophilic parts into the bulk solution. 

There are also several investigations focused on myoglobin adsorption on various nanoparticles [31,32], which leads to soft and hard corona formation. 

However, this analysis of literature data indicates that there are few systematic investigations focused on measurements of myoglobin adsorption kinetics at substrates of well-defined surface properties and morphology, which can yield reliable information about plausible mechanisms of this process. 

Therefore, in this work we performed such measurements by applying quartz crystal microbalance (QCM) combined with optical waveguide light mode spectroscopy (OWLS) methods, which facilitated in situ and real-time investigations. Additionally, the morphology of the adsorbed protein layers was determined by ex situ AFM investigations. A quantitative analysis of the kinetic adsorption and desorption runs enabled to determine the maximum coverage of irreversibly and reversibly bound myoglobin molecule fractions. Also, the equilibrium adsorption constant that characterized the binding energy of reversibly bound molecules was acquired. It is expected that our investigations furnish valid information about the adsorption mechanism of myoglobin molecules. This knowledge can be exploited to develop a procedure for preparing myoglobin layers at silica substrates of well-controlled coverage that can be used for biosensing purposes. 

## 2. Materials and Methods 

In our investigations, myoglobin stemming from equine skeletal muscles supplied in the form of a lyophilized powder 95–100% (Sigma-Aldrich) was used. 

Other chemical reagents such as sodium chloride and hydrochloric acid were commercial products of Sigma-Aldrich and were used without additional purification. Ultrapure water was obtained using the Milli-Q Elix&Simplicity 185 purification system from Millipore. 

The concentration of myoglobin, after dissolving the powder in appropriate electrolyte and pH and after filtration, was spectrophotometrically determined using a Shimadzu UV-2600 apparatus exploiting the peak absorption at 409 nm.

In the electrophoretic mobility and the dynamic light scattering measurements, carried out using the Zetasizer Nano ZS instrument of Malvern, stock solutions characterized by myoglobin concentration equal to 300–500 mg L^−1^ were used. For performing the adsorption kinetics experiments, the stock solutions were diluted to the desired bulk concentration, typically 5–10 mg L^−1^. Levels of pH in the range of 3–9 were adjusted by the addition of either HCl or NaOH, whereas a pH of 7.4 was fixed by the PBS buffer.

From the electrophoretic mobility data, the zeta potential of myoglobin molecules was calculated using the Henry formula. 

On the other hand, the zeta potential of silica as a function of pH and for different ionic strengths was determined by the streaming potential measurements performed in a microfluidic four-electrode cell [33]. The Smoluchowski model was used in order to calculate the zeta potential from the experimentally acquired streaming potential. 

The quartz microbalance (QCM) measurements of myoglobin adsorption kinetics were carried out according to the procedure previously applied in ref. [34] using the quartz/silicon dioxide (SiO_2_) sensors supplied by QSense, Gothenburg, Sweden. The sensors were cleaned before each experiment in a 30-minute-old mixture of 96% sulfuric acid (H_2_SO_4_), hydrogen peroxide (30%) and pure water in the volume ratio 1:1:1 for 2 min. Afterward, the sensors were rinsed by deionized water at 80 °C for 30 min and dried out in a gentle stream of nitrogen gas. 

The coverage of myoglobin was calculated from the Sauerbrey equation [34,35].

(1) $$\Gamma_{Q}=-C_{Q}\frac{\Delta f}{n_{0}}$$

where $\Gamma_{Q}$ is the protein coverage, *C_Q_* is the Sauerbrey constant equal to 0.177 mg m^−2^ Hz^−1^ for the 5 MHz AT‑cut quartz sensor [34,35], Δf is the frequency change and *n*_0_ is the overtone number. 

After completing the adsorption/desorption run, myoglobin molecules on silica sensors were imaged using ambient air atomic force microscopy (AFM), NT-MDT Solver BIO device with the SMENA SFC050L scanning head. 

The optical waveguide light-mode spectroscopy (OWLS) measurements of myoglobin adsorption kinetics were carried out using the Microvacuum Ltd., (Budapest, Hungary) instrument equipped with a flow cell comprising a silica-coated waveguide (OW 2400, Microvacuum). The adsorbing substrates were planar optical waveguides (OW 2400 from MicroVacuum, Budapest, Hungary) made of glass (refractive index 1.526) covered by a film of Si_0.78_Ti_0.22_O_2_ with a thickness of 170 nm, and with a refractive index of 1.8. A grating embossed in the substrate enabled the light to be coupled into the waveguide layer. The sensor surface was coated with an additional layer (10 nm) of pure SiO_2_ [36]. Adsorption to the waveguide surface alters the interfacial refractive index and, therefore, the in-coupling angles of the laser light was monitored. Assuming an optically uniform adsorbed layer, the mass of adsorbed protein can be calculated from the Feijter formula [37] 

All measurements were performed at 298 K.

## 3. Results and Discussion 

### 3.1. Physicochemical Characteristics of Myoglobin Molecules and Silica 

The diffusion coefficient and the electrophoretic mobility of myoglobin molecules were determined using the Dynamic Light Scattering (DLS) and the Laser Doppler Velocimetry (LDV) methods, respectively. The average diffusion coefficient for the pH range 3–8 was equal to 1.2 ± 0.1 × 10^−6^ cm^2^ s^−1^. This corresponds to the hydrodynamic diameter of the myoglobin molecules $d_{H}$ calculated from the Stokes–Einstein relationship equals to 4.1 ± 0.2 nm. 

**Figure 1:**
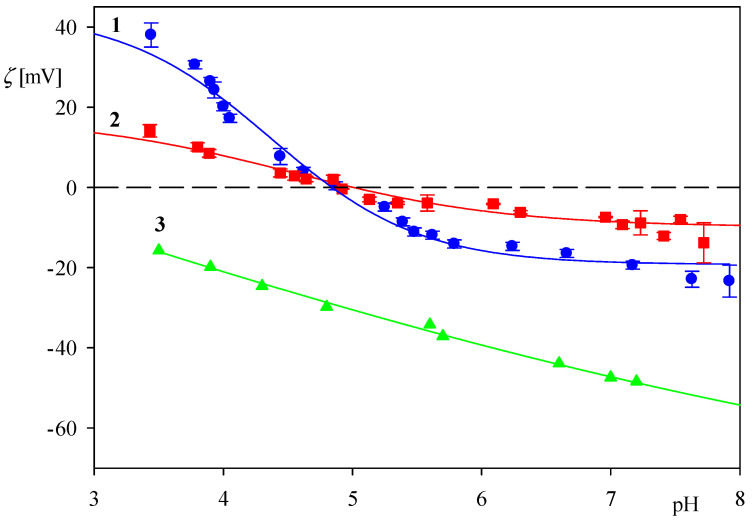
Dependence of the zeta potential on pH for: 1. myoglobin molecules in 0.01 M, NaCl (LDV measurements), 2. myoglobin molecules in 0.15 M, NaCl (LDV measurements), 3. silica in 0.01 M, NaCl (the streaming-potential measurements).

The dependence of the zeta potential of myoglobin molecules on pH for NaCl concentrations equal to 0.01 and 0.15 M is presented in Figure 1. As can be seen, it assumes positive values at pH < 5 and approaches 38 and 15 mV at pH 3.5, for NaCl concentration of 0.01 and 0.15 M, respectively. At pH > 5, the zeta potential becomes negative, approaching −20 and −10 mV, at pH 7.4 (PBS) for NaCl concentration of 0.01 M and 0.15 M, respectively. It should be observed, however, that for the pH range 4.5–6, the zeta potential assumes small values, especially for the 0.15 M NaCl concentration, comparable with the experimental error bounds. Therefore, a precise determination of the myoglobin isoelectric point (iep) is rather difficult. 

On the other hand, the zeta potential of oxidized silicon wafers was determined by the streaming potential measurements carried out according to a procedure described elsewhere [38]. As can be seen in Figure 1 at pH 3.5 and 7.4 the wafer zeta potential is equal to −15 and −50 mV, respectively. It is assumed that that the zeta potential of the silica sensor used in the QCM investigations is equal to the above data pertinent to the Si/SiO_2_ wafer. 

The surface topography, comprising the roughness of the QCM sensors, was determined by atomic force microscopy (AFM) carried out in a semi-contact mode under ambient conditions. It was confirmed that the sensors were characterized by the root mean square roughness equal to 0.90 nm. It should be mentioned that in contrast to ref. [29] the skewness and surface kurtosis of the substrate surfaces were not determined because of a rather large tip radius exceeding 10 nm. 

### 3.2. The Kinetics of Myoglobin Adsorption

**Figure 2:**
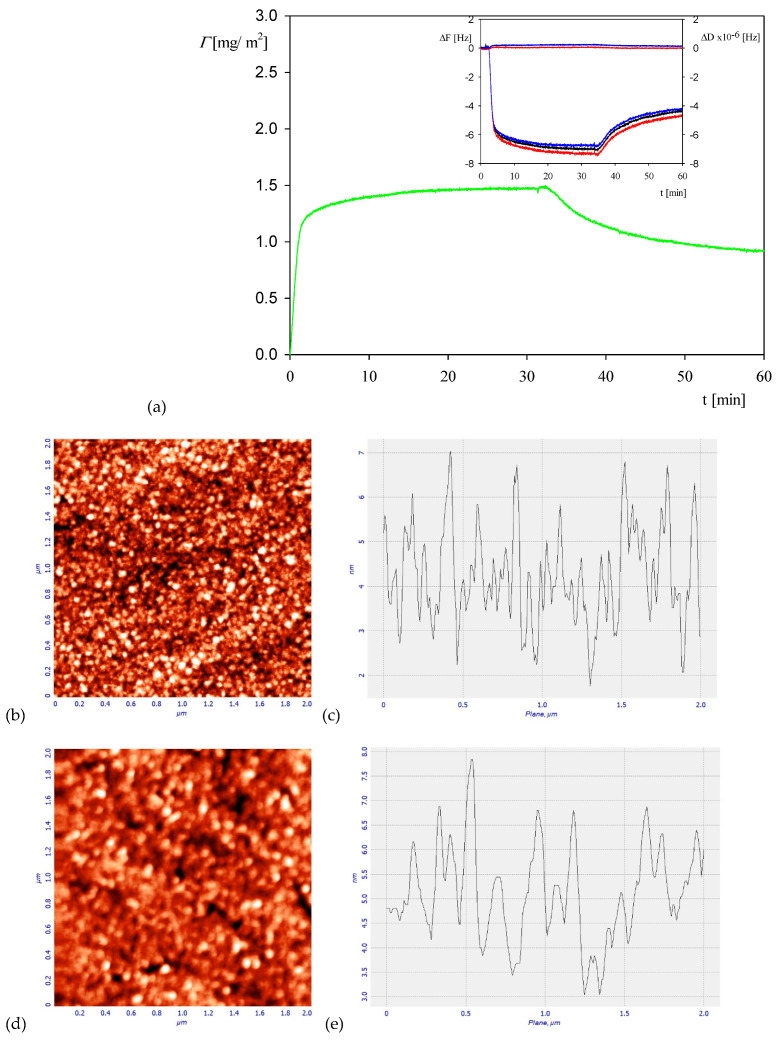
Part (**a**) the kinetics of myoglobin adsorption on silica sensor determined by QCM, flow rate 2.5 × 10^−3^ cm^3^ s^−1^, bulk protein concentration equal to 5 mg L^−1^, 0.01 M NaCl, pH 3.5. At the time of 30 min, the rinsing run was initiated by flushing pure electrolytes at the same ionic strength and pH. The inset shows the primary frequency shift ΔFq (left hand axis) and dissipation ΔD (right hand axis) as a function of time at the following overtones: ●—7th, ●—9th, ●—11th. Part (**b**) the AFM image of the silica sensor with adsorbed myoglobin molecules, part (**c**) the height profile of the protein layer adsorbed at the sensor. Part (**d**) the AFM image of the clean silica sensor, part (**e**) the height profile of the clean silica sensor.

A representative QCM adsorption/desorption kinetic run performed for the myoglobin bulk concentrations of five mg L^−1^, NaCl concentration of 0.01 M and pH of 3.5 is shown in Figure 2. 

One can observe that the protein coverage (calculated from Equation (1) as an average from the third to seventh overtones) rapidly increases in a linear way attaining after the time of 30 min a plateau value equal to 1.5 mg m^−2^. Afterward, upon flushing with pure electrolyte solution at the same flow rate, a significant decrease in the coverage is observed yielding a stationary value equal to 1 mg m^−2^, which corresponds to irreversibly bound molecule fraction. This is illustrated in Figure 2b, which shows the AFM image of the myoglobin layer at the silica sensor with a typical height profile. However, one should mention that due to the roughness of the silica sensor, a quantitative evaluation of the myoglobin molecule coverage is not feasible. 

It should be pointed out that the protein coverage derived from QCM measurements does not correspond to the true physical (dry) coverage, defined as the mass of adsorbed protein molecules per unit area, because of the hydrodynamic coupling effects previously observed for other proteins [34,36]. Therefore, the physical (dry) coverage Γ can be calculated as

(2) $$\Gamma =\Gamma_{Q}(1-H)$$

where $\Gamma_{Q}=\Gamma +\Gamma_{s}$ is the protein coverage derived from QCM measurements, $\Gamma_{s}$ is the coupled solvent coverage (mass) and *H* is the hydration function [34,35,39]. 

The hydration function can be determined as described in ref. [34] using the solution of the mass transfer equation governing protein molecule transport to the sensor. It is shown that under the convective diffusion regime, the adsorbed protein coverage is described by the linear relationship

(3) $$\Gamma =k_{c}c_{b}t$$

where *k_c_* is the mass transfer rate constant and *c_b_* is the bulk concentration of a protein. 

One should mention that Equation (3) is valid for a broad range of protein and nanoparticle coverage slightly below the maximum coverage [40]. 

The *k_c_* constant can be calculated either via numerical solutions of the mass transfer equation considering the flow pattern in the QCM cell, or more convenient performing calibrating deposition kinetic measurements using nanoparticles characterized by a large density. In this way, it was determined in ref. [41] using gold nanoparticles that the $k_{c}$ constant for the QCM cell used in this work is given by

(4) $$k_{c}=C_{f}Q^{\frac{1}{3}}D^{\frac{2}{3}}$$

where *C_f_* = 19 cm^−2/3^ is the constant depending on the cell geometry, *Q* is the volumetric flow rate of the protein solution and *D* is the diffusion coefficient of protein molecules. 

For myoglobin, using the above diffusion coefficient, one obtains *k_c_* = 2.9 × 10^−3^ L m^−2^ s^−1^ = 2.9 × 10^−4^ cm s^−1^ for the flow rate of 2.5 × 10^−3^ cm^3^ s^−1^. 

Using Equation (2) one can express the hydration function in the following form

(5) $$H=1-\frac{k_{c}c_{b}t}{\Gamma_{Q}(t)}$$

where $\Gamma_{Q}(t)$ is the time- dependent coverage derived from QCM measurements. 

**Figure 3:**
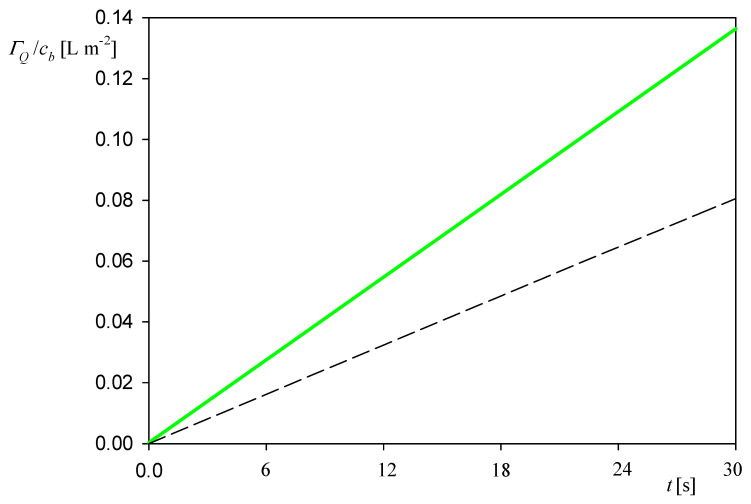
Dependence of the normalized myoglobin coverage *Γ_Q_*/*c_b_* on the adsorption time *t.* Results are averaged from five independent runs performed at different bulk suspension concentrations (5 and 10 mg L^−1^); 0.01 M NaCl, flow rate of 2.5 × 10^−3^ cm^3^ s^−1^, pH 3.5 and 4. The dashed line shows the dry mass calculated from Equation (3).

Such kinetic runs obtained for different bulk protein concentrations are plotted in Figure 3 as the dependence of the normalized coverage *Γ_Q_*/*c_b_* on the adsorption time *t*. One can see that these results can be fitted by a linear dependence in respect to the adsorption time given by

(6) $$\Gamma_{Q}/c_{b}=k_{Q}t$$

where $k_{Q}$ is equal to 4.4 ± 0.1 × 10^−3^ L m^−2^ s^−1^ = 4.4 ± 0.1 × 10^−4^ cm s^−1^. 

The applicability of Equation (5) confirms that the hydration function of myoglobin layers was independent of the coverage; therefore, the hydration function assumes the form

(7) $$H=1-\frac{k_{c}}{k_{Q}}$$

Therefore, by virtue of Equation (3), the dry protein coverage can be calculated as

(8) $$\Gamma_{}=\Gamma_{Q}\frac{k_{c}}{k_{Q}}$$

Using the previously determined value of *k_c_* = 2.9 × 10^−3^ L m^−2^ s^−1^ one can calculate from Equation (7) that *H* = 0.34. For comparison, in the case of albumin (HSA) adsorption at the silica sensor, the hydration function in the limit of low coverage was equal to 0.65 at pH 3.5 and 0.01 M NaCl concentration [42]. Given that the hydrodynamic diameter of the myoglobin molecule equal to 4.1 nm it is much smaller than the one of the HSA molecule, equal to 7.9 nm [43] the lower value of the hydration function can be attributed to the sensor roughness effect discussed in ref. [43]. 

The QCM kinetic runs shown in Figure 2 was also thoroughly analyzed in respect to the desorption regime in order to determine the reversibly bound protein fraction, the equilibrium adsorption constant and the binding energy. One can see that during the desorption the coverage decreased from 1.5 mg m^−2^ to 0.9 mg m^−2^ after the time of 30 min. Using the above estimated value of the hydration function, one can calculate from Equation (3) that the dry coverage of irreversibly bound myoglobin is equal to 0.60 ± 0.1 mg m^−2^ (at 0.01 M NaCl and a pH of 3.5–4). It is interesting to mention that for albumin (HSA) the coverage was equal to 0.7 mg m^−2^ under the same pH and NaCl concentration [43]. 

**Figure 4:**
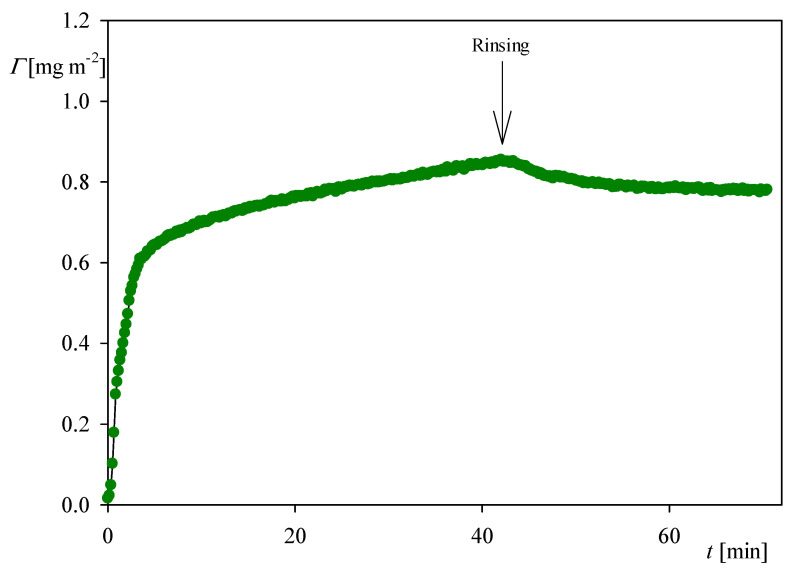
The kinetic of myoglobin adsorption on the silica sensor determined by OWLS, flow rate 2.5 × 10^−3^ cm^3^ s^−1^, bulk protein concentration equals to 5 mg L^−1^, ionic strength 0.01 M, pH 4. At the time of 45 min the rinsing run was initiated by flushing pure electrolyte at the same ionic strength and pH.

It is interesting to compare the QCM kinetics with that derived from OWLS measurements, which is presented in Figure 4. It can be seen that the kinetic is fully analogous to that recorded by QCM (see Figure 2a), i.e., it is characterized by a rapid and linear increase in the coverage for the time up to five minutes. Afterward, the coverage increases at a much smaller rate and, upon flushing with pure electrolyte solution at the same flow rate, the coverage decreases to 0.7 mg m^−2^ due to the desorption of weakly bound molecules. This stationary coverage agrees within error bounds with the QCM result. 

On the other hand, the myoglobin desorption kinetics can be quantitatively analyzed using the method developed in ref. [13]. The equation describing protein desorption kinetics under convective-diffusion transport can be approximated by the formula

(9) $$\Gamma_{r}(t)=\Gamma_{r0}{}_{}^{}e^{-\frac{k_{c}}{B_{0}K_{a}}t}$$

where $\Gamma_{r}(t)$, $\Gamma_{r0}$ are the time dependent and the initial coverage of reversibly bound protein fractions, *B*_0_ is the blocking constant for the reversibly bound molecule fraction and *K_a_* is the equilibrium adsorption constant. 

From Equation (9) one can deduce that *K_a_* can be determined from the formula

(10) $${Κ}_{a}=-\frac{k_{c}}{B_{0}s_{l}}$$

where *s_l_* is the slope of the dependence of $\ln[\Gamma_{r}(t)/\Gamma_{r0}]$ on the desorption time *t_d_*. 

**Figure 5:**
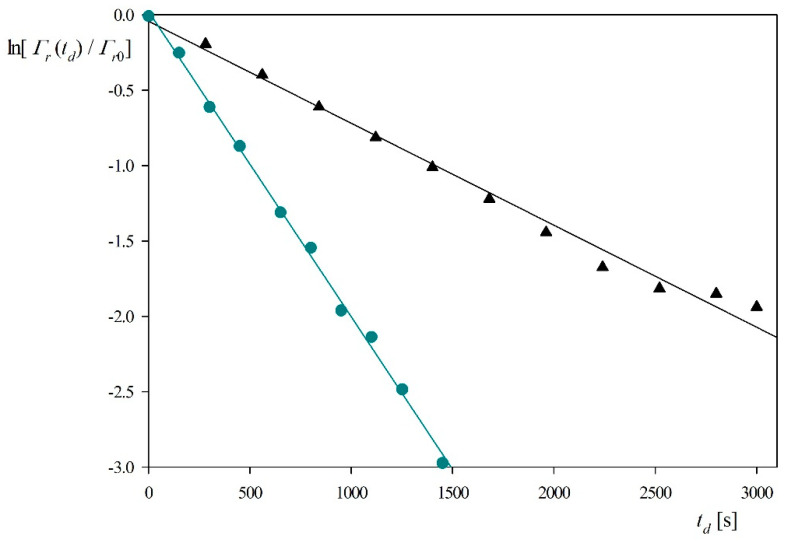
Myoglobin desorption kinetics derived from QCM and expressed as the dependence of $\ln[\Gamma_{r}(t_{d})/\Gamma_{r0}]$ on the desorption time *t_d_*; silica sensor, pH 3.5 (triangles), pH 4 (circles), NaCl concentration 0.01 M, flow rate 2.5 × 10^−3^ cm^3^ s^−1^. The lines represent linear fits of experimental data.

It should be observed that the ratio $\Gamma_{r}(t)/\Gamma_{r0}$ is equal to $\Gamma_{QCMr}(t)/\Gamma {\;}_{QCMr0}$ because the hydration function is constant for this coverage range. The dependencies of $\ln[\Gamma_{r}(t)/\Gamma_{r0}]$ on the desorption time obtained for pH 3.5 and 4 (0.01 M NaCl, flow rate 2.5 × 10^−3^ cm^3^ s^−1^) are shown in Figure 5. One can observe that the experimental data can be fitted by a straight line characterized by the slopes equal to −6.8 × 10^−4^ and −2.0 × 10^−3^ s^−1^ for pH 3.5 and 4, respectively. Considering that *k_c_* = 2.9 × 10^−4^ cm s^−1^ and assuming *B*_0_ = 0.5, one can calculate from Equation (9) that *K_a_* = 0.85 and 0.29 cm for pH equal to 3.5 and 4, respectively. The decrease in the adsorption constant can be interpreted as due to a rapid decrease in the zeta potential of myoglobin molecules with pH (see Figure 1). 

Knowing *K_a_* one can determine the binding energy (energy minimum depth) $\phi_{m}$ from the following equation derived in ref. [13]

(11) $$\phi_{m}/kT+\frac{1}{2}\ln\left(\frac{\left|\phi_{m}\right|}{\pi kT}\right)=\ln(\delta_{m}/K_{a})$$

where 2*δ_m_* is the energy minimum width.

Using the above value of the *K_a_* constant and assuming 2*δ_m_* = 3 nm, one obtains from Equation (11) that the binding energy for the reversibly adsorbed myoglobin molecules is equal to −16.5 and −15.5 *kT* for pH 3.5 and 4, respectively. 

It is interesting to mention that similar values of *K_a_* = 1.2 cm and $\phi_{m}$ equal to −17.8 *kT* were previously determined in ref. [43] for the human serum albumin (HSA) desorption from silica sensor (at pH 3.5, 0.01 M NaCl concentration) applying an exact solution of the mass transfer equation. 

**Figure 6:**
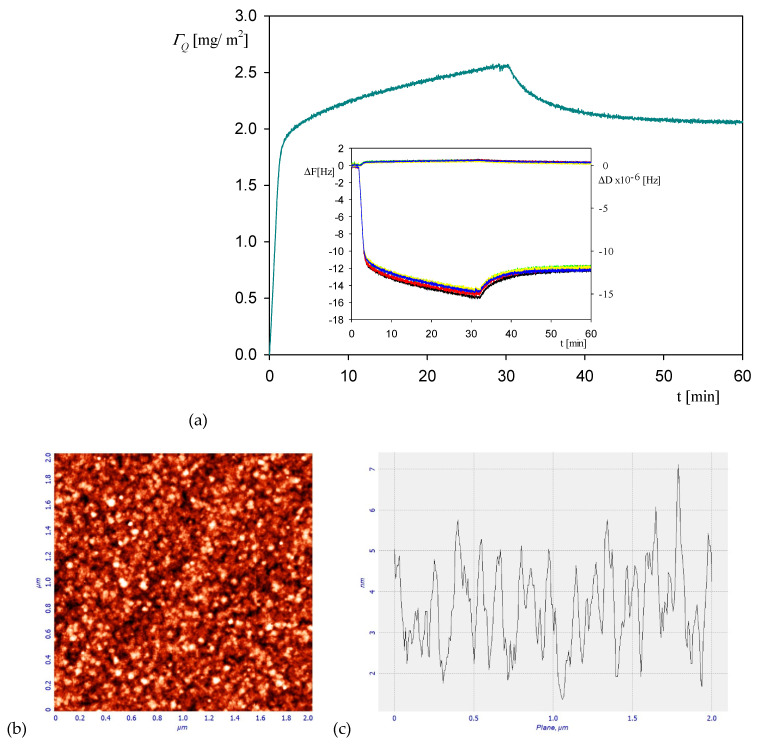
Part (**a**) the kinetic of myoglobin adsorption on silica sensor determined by QCM, flow rate 2.5 × 10^−3^ cm^3^ s^−1^, bulk protein concentration equal to 10 mg L^−1^, 0.15 M NaCl, pH 3.5. At the time of 35 min the rinsing run was initiated by flushing pure electrolyte at the same ionic strength and pH. The inset shows the primary frequency shift ΔFq (left hand axis) and dissipation ΔD (right hand axis) as a function of time at the following overtones: ●—3rd, ●—5th, ●—7th, ●—9th, ●—11th. Part (**b**) the AFM image of the silica sensor with adsorbed myoglobin molecules. Part (**c**) the height profile of the protein layer adsorbed at the sensor.

Analogous kinetic measurements were performed for the NaCl concentration of 0.15 M, with the aim of determining the influence of ionic strength on the maximum coverage of irreversibly bound protein. A typical QCM run shown in Figure 6a confirms that the protein coverage linearly increases with the adsorption time attaining the maximum value of 2.5 mg m^−2^ at after the time of 30 min. Afterward, upon flushing with pure electrolyte solution at the same flow rate, the coverage monotonically decreases, attaining a stationary value equals to 2 mg m^−2^. The AFM image of the myoglobin layer at the silica sensor after completing the desorption run is shown in Figure 6b. Using this stationary coverage and the above estimate of the hydration function, one obtains 1.3 ± 0.1 mg m^−2^ as a plausible value of the dry coverage, which is more than two times larger than that for 0.01 M NaCl. For comparison, the dry coverage of irreversibly bound albumin at silica sensor at pH 3.5 and 0.15 M NaCl determined in ref. [44] from QCM measurements was equal to 1.4 ± 0.05 mg m^−2^, which agreed with the experimental data obtained for HSA adsorption at mica using the streaming potential method [45]. 

It should be mentioned that analogous kinetic runs, as shown in Figure 6, were acquired for pH 4 characterized by the same maximum coverage within experimental error bounds. 

**Figure 7:**
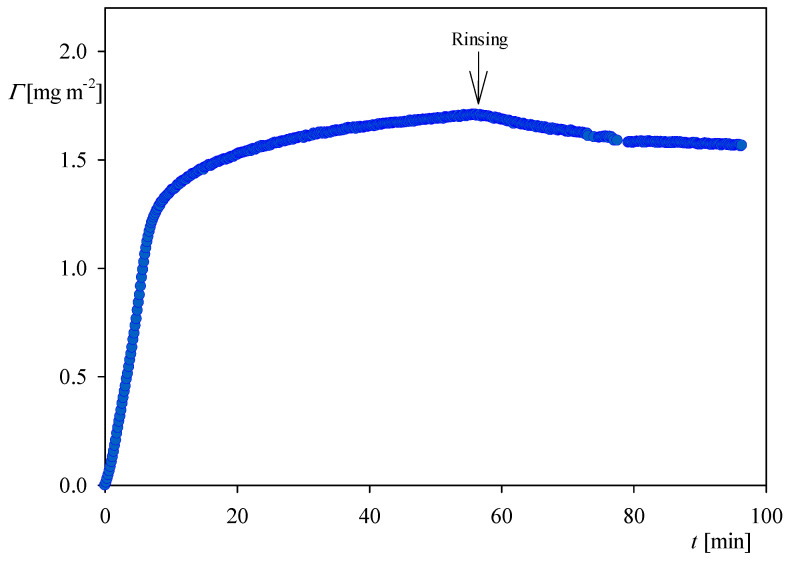
The kinetics of myoglobin adsorption on the silica sensor determined by OWLS, flow rate 2.5 × 10^−3^ cm^3^ s^−1^, bulk protein concentration equal to 5 mg L^−1^, ionic strength 0.15 M, pH 4. At the time of 50 min the rinsing run was initiated by flushing pure electrolyte at the same ionic strength and pH.

Analogous measurements derived from OWLS for NaCl concentration of 0.15 M presented in Figure 7 indicate that the stationary coverage of myoglobin after the desorption was equal to 1.5 mg m^−2^, which is also two times larger than for an NaCl concentration of 0.01 M. 

This considerable increase in the maximum coverage of myoglobin with the NaCl concentration (ionic strength) can be interpreted in terms of the decreased range of lateral electrostatic interactions among adsorbed molecules, which are governed by the electric double-layer thickness [45,46]. Indeed, for 0.01 NaCl concentration, the double-layer thickness is equal to 3.1 nm, which exceeds the radius of the myoglobin molecule, whereas for 0.15 M NaCl the double-layer thickness decreases to 0.8 nm, which is more than two times smaller than the molecule radius. Therefore, in the latter case, the lateral repulsion among adsorbed molecules is practically eliminated and the maximum coverage should attain the value pertinent to the non-interacting molecules approximated as hard spheres. The maximum coverage of myoglobin calculated from the random sequential adsorption (RSA) model for hard spheres was equal 1.2 to 1.5 mg m^2^, for the molecule cross-section area of 11 to 13 nm^2^, respectively [46]. This comparison confirms that the electrostatic interactions play a significant role and control the maximum coverage of irreversibly adsorbed molecules.

**Figure 8:**
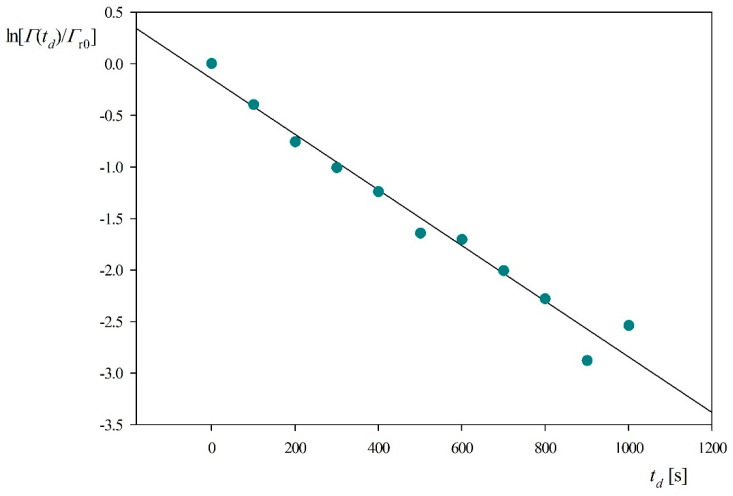
Myoglobin desorption kinetics derived from QCM and expressed as the dependence of $\ln[\Gamma_{r}(t_{d})/\Gamma_{r0}]$ on the desorption time *t_d_*; silica sensor pH 3.5, NaCl concentration 0.15 M, flow rate 2.5 × 10^−3^ cm^3^ s^−1^.

Electrostatic interactions are also expected to affect the myoglobin molecule desorption rates. This can be quantitatively analyzed plotting as previously the dependence of $\ln[\Gamma_{r}(t_{d})/\Gamma_{r0}]$ on the desorption time, see Figure 8. One can observe that the experimental data obtained for 0.15 M, NaCl concentration (pH 3.5) can be fitted by a straight line characterized by the slope equal to −2.7 × 10^−3^ s^−1^. Considering that *k_c_* = 2.9 × 10^−4^ cm s^−1^ and assuming *B*_0_ = 0.5 one can calculate from Equation (10) that *K_a_* = 0.21 cm compared to the previous value of 0.85 cm obtained for 0.01 M NaCl and pH 3.5. Using the above value of the *K_a_* constant and assuming 2*δ_m_* = 0.8 nm one obtains from Equation (11) that the binding energy for the reversibly adsorbed myoglobin molecules is equal to −16 *kT*. These results indicate that indeed the increased ionic strength caused a decrease in the equilibrium adsorption constant and the molecule binding strength. 

**Figure 9:**
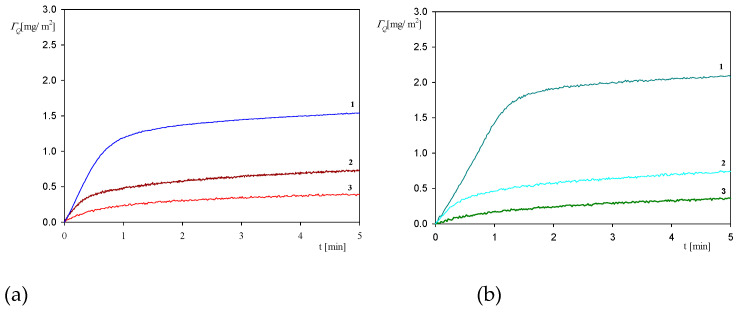
The kinetics of myoglobin adsorption on the silica sensor determined by QCM, flow rate 2.5 × 10^−3^ cm^3^ s^−1^, bulk protein concentration equal to 10 mg L^−1^. Part (**a**); ionic strength 0.01 M, 1. pH 3.5, 2. pH 5.5, 3. pH 7.4. Part (**b**); ionic strength 0.15 M, 1. pH 3.5, 2. pH 5.5, 3. pH 7.4.

In order to investigate the role of electrostatic interactions, a further series of measurements were performed using the QCM method at larger pHs, equal to 5.5 and 7.4. The results shown in Figure 9 confirm that the myoglobin adsorption kinetics at both pHs is considerably less efficient compared to pH 3.5, both in respect to the initial rate and the maximum coverage attained after longer adsorption time. Thus, at pH 5.5, the myoglobin coverage after the adsorption time of five minutes is four times smaller, and for pH 7.4 almost ten times smaller compared to pH 3.5. These experimental data can be qualitatively interpreted considering the dependencies shown in Figure 1 where one can observe that at a pH larger than five the myoglobin molecule zeta potential vanishes and then becomes negative at pH 7.4. Therefore, it is expected that the electrostatic interactions of myoglobin molecules with the negatively charged sensor should be much weaker at these pHs, prohibiting stronger adhesion contact of single molecules to be formed. 

However, given that a measurable adsorption of myoglobin at pH 5.5 and 7.4 is observed, one can suppose that the sensor and the protein molecule charge heterogeneity can play a significant role. Because however, a quantitative interpretation of this effect requires detailed information about the morphology of the sensor, its chemical composition and charge distribution in the nanoscale, which is not currently not available. Another plausible explanation of the measurable, albeit much slower, myoglobin adsorption at pH 5.5 and 7.4 compared to pH 3.5 may consist in the protein solution aggregation, which was quantitatively analyzed in ref. [46] and extensively discussed in the review [47]. 

## 4. Conclusions

It is shown that adsorption and desorption kinetics of myoglobin on the silica sensor at different ionic strengths and pHs under flow conditions can be determined by applying the QCM and the OWLS measurements. A quantitative analysis of the experimental runs enabled to determine the maximum coverage of irreversibly bound myoglobin molecule. At a pH of 3.5, this was equal to 0.60 and 1.3 mg m^−2^ for a NaCl concentration of 0.01 and 0.15 M, respectively. The latter value agrees with the maximum coverage pertinent to a closely packed monolayer of molecules predicted from the random sequential adsorption model. It is argued that these results confirm that lateral interactions among adsorbed protein molecules stemming from the presence of electric double layers play a significant role. 

Additionally, a proper interpretation of the desorption runs induced by electrolyte rinsing furnished a valid estimate of the equilibrium adsorption constant and the binding energy of the reversibly bound molecules.

It is also confirmed that at a pH above five, the adsorption of myoglobin was considerably less effective. This was attributed to the vanishing net charge of molecules that decreased their binding energy with the substrate. A residue adsorption of myoglobin at pH 5.5 and 7.4 was interpreted in terms of the sensor charge heterogeneity.

Beside significance to basic science, these results can be exploited to develop a reliable procedure for preparing myoglobin layers of well-controlled coverage, useful for biosensing purposes. 

## Data Availability

The data presented in this study are available on request from the corresponding author.
